# Targeted tissue perfusion versus macrocirculation-guided standard care in patients with septic shock (TARTARE-2S): study protocol and statistical analysis plan for a randomized controlled trial

**DOI:** 10.1186/s13063-016-1515-x

**Published:** 2016-08-02

**Authors:** Ville Pettilä, Tobias Merz, Erika Wilkman, Anders Perner, Sari Karlsson, Theis Lange, Johanna Hästbacka, Peter Buhl Hjortrup, Anne Kuitunen, Stephan M. Jakob, Jukka Takala

**Affiliations:** 1 Department of Intensive Care Medicine, Bern University Hospital (Inselspital), University of Bern, Bern, Switzerland; 2 Division of Intensive Care Medicine, Department of Perioperative, Intensive Care and Pain Medicine, University of Helsinki and Helsinki University Hospital, Helsinki, Finland; 3 Department of Intensive Care, Copenhagen University Hospital, Rigshospitalet, Copenhagen, Denmark; 4 Department of Intensive Care, Tampere University Hospital, Tampere, Finland; 5 CRIC, Center of Research for Intensive Care, Copenhagen, Denmark; 6 Section of Biostatistics, University of Copenhagen, Copenhagen, Denmark

**Keywords:** Septic shock, Critical illness, Tissue perfusion, Lactate, Vasopressor, Mortality

## Abstract

**Background:**

Septic shock has a 90-day mortality risk of up to 50 %. The hemodynamic targets, including mean arterial pressure (MAP) are not based on robust clinical data. Both severe hypotension and high doses of vasopressors may be harmful. Hence, re-evaluation of hemodynamic targets in septic shock is relevant.

**Methods/design:**

The targeted tissue perfusion versus macrocirculation-guided standard care in patients with septic shock (TARTARE-2S) trial is a prospective, two-parallel-group, randomized, open-label, multicenter trial with assessor-blinded outcome evaluation. We will randomize at least 200 patients with septic shock in four European intensive care units (ICUs) to test whether a tissue perfusion-guided treatment strategy based on capillary refill time, peripheral temperature, arterial lactate concentrations, and accepting lower MAP levels, leads to a faster resolution of shock than macrocirculation target-guided standard care.

The primary outcome measure is days alive in 30 days with normal arterial blood lactate (first value of <2 mmol/L) and without any inotropic or vasopressor agent. Secondary outcomes include individual components of the primary outcome, days alive without renal replacement, days alive without mechanical ventilation in 30 days, and new acute kidney injury. The sample size enables detection of a 13.5-h difference in the primary outcome with a type 1 error of 5 % and power of 80 %, assuming 25 % mortality and a mean of 650 h (SD 30) among the 30-day survivors. After 150 included patients the statistician masked for allocation group will recalculate the sample size potentially increasing the sample up to 300. The Data Safety and Monitoring Board (DSMB) will review the safety data after 100 patients.

**Discussion:**

The TARTARE-2S trial will provide important clinical data on treatment targets in septic shock, evaluating the impact of clinical tissue perfusion-guided hemodynamic treatment on a surrogate outcome combining resolution of shock (hyperlactatemia and vasopressors/inotropes), and 30-day mortality.

**Trial registration:**

ClinicalTrials.gov: NCT02579525. Registered on 19 October 2015.

**Electronic supplementary material:**

The online version of this article (doi:10.1186/s13063-016-1515-x) contains supplementary material, which is available to authorized users.

## Background

Septic shock affects millions of people annually and has a 90-day mortality rate of 18 to 50 % [1–5] depending on the patient population and study selection criteria. Early hemodynamic resuscitation and stabilization is considered to be crucial for improved survival. Current resuscitation strategies aim to achieve hemodynamic stabilization as early as possible in order to prevent subsequent organ dysfunction. The guidelines for initial hemodynamic resuscitation of septic shock use primarily macrocirculatory targets, such as mean arterial blood pressure (MAP) and central venous pressure (CVP), and surrogate markers of tissue perfusion such as diuresis. Attempts to use central venous or mixed venous oxygen saturation (ScVO_2_, SvO_2_) to guide resuscitation have been disappointing. Despite early enthusiasm based on a small single-center trial which suggested a major improvement in survival [6], no benefit of ScVO_2_-guided treatment was found in three large multicenter trials [1, 4, 5].

The current SSC guidelines (Surviving Sepsis Campaign: International Guidelines for Management of Severe Sepsis and Septic Shock: 2012) [7] still emphasize a MAP target and to a lesser extent tissue perfusion. Systemic blood pressure and flow and microcirculatory blood flow do not correlate well in septic shock. This is especially true if systemic blood pressure is achieved with vasoconstrictive drugs which per se may impair microcirculation and clinical outcome [8]. Furthermore, a recent systematic review [9] underscored the paucity of clinical evidence to guide the use of vasopressors and to support the current recommendations regarding MAP target levels. In summary, the current resuscitation guidelines for septic shock might direct physicians to use higher doses of vasopressors and inotropes and, thus, facilitate iatrogenic aggravation of shock-induced tissue hypoperfusion.

Impaired tissue perfusion and microcirculation are considered to be hallmarks of septic shock, and increased arterial blood lactate in septic shock is a marker of poor prognosis. These concepts provide a rationale for therapeutic strategies focusing more on improvement of tissue perfusion than systemic hemodynamics. Normalization of elevated blood lactate levels has been proposed to guide resuscitation in septic shock because it may reflect tissue perfusion during shock resuscitation better than strategies based mainly on macrohemodynamic targets. The duration of elevated lactate levels is a clinically relevant surrogate endpoint previously related to robust patient-related adverse outcomes, such as multiple organ dysfunction [10] and mortality [11, 12]. In addition, lactate is also associated with peripheral perfusion [13], and has been used as a target in a previous large study in septic shock in comparison to ScVO_2_-targeted therapy [14]. Abnormal peripheral perfusion, defined as arm-to-fingertip temperature gradient of >4 °C, and capillary refill time (CRT) >4.5 s), is associated with multiple organ dysfunction in critically ill patients [15] and with impaired tissue oxygen saturation and poor outcome in septic shock patients [16]. Of note, subjectively assessed warmness of the peripheral skin has good agreement with the measured temperature differences [15]. The mottling score [17], reflecting perfusion of the knee skin area, has also been used. Additionally, biomarkers (to be explored in sub-studies) indicating endothelial or cardiac damage could elucidate the potential mechanisms leading to untoward clinical patient-related outcomes, such as septic acute kidney injury (AKI) [18] treated with renal replacement therapy (RRT) [19], multiple organ dysfunction, and death.

### Aim

The objective is to compare the feasibility and the effect of resolution of shock of two approaches to the management of tissue hypoperfusion in septic shock: (1) targeted tissue perfusion (TTP) approach versus (2) macrocirculation-guided (MCG) care – the latter reflecting recommended standard care.

## Methods/design

This trial is a prospective, investigator-initiated, multicenter, two-parallel-group, randomized, open-label, multicenter trial of TTP versus MCG care in patients with septic shock (Appendix 1) with adequate generation of allocation sequence, and adequate allocation concealment. Patients will be stratified by center and by the presence/absence of chronic hypertension. Each participating unit will go through an educational program regarding study monitoring methods (including CRT), and study reporting methods (including repeated hemodynamic target assessments and treatment attempts).

The recruitment begins after approval from local ethics committees.

### Ethics committee approvals submission/approval

Bern University Hospital Ethics Committee (Kantonal Ethiko Kommittee (KEK) Bern) 19 October 2015/22 March 2016.

Helsinki University Hospital Operative Ethics Committee – 23 October 2015/16 December 2015.

Rigshospitalet Ethics Committee – April 2016/pending.

### Hypothesis

We hypothesize that targeting clinical tissue perfusion (the TTP arm) will decrease the use and untoward effects of vasopressors, and result in more days alive in 30 days with normal arterial blood lactate (first value of <2 mmol/L) *and* without any inotropic or vasopressor agent – compared to standard clinical care with preference of macrocirculatory targets (the MCG arm).

### Trial interventions

All patients will be treated according to the targets (Appendix 2) of the allocated arm:

1. Intervention group – targeted tissue perfusion (TTP) care
2. Control group – macrocirculatory targets-guided (MCG) standard care

All interventions in both groups will be given at the discretion of the treating clinicians according to the targets. Both the hemodynamic problems detected and the given interventions will be registered at each change of treatment over time (Appendices 3 and 4).

### Concomitant interventions

Treatment of septic shock is complex with multiple interventions [6] and, as blinding of treating personnel is not feasible, the use of several concomitant interventions may be influenced by the allocated intervention arm. In order to minimize these potential differences, treatment suggestions for the following interventions will be provided:

- Vasopressors – norepinephrine highly recommended (the hemodynamic problems to be registered and reported – Appendix 3)
- Fluids – correction of hypovolemia (preferably crystalloids, starch not to be used) will be at the discretion of the treating clinician (Appendix 3)
- Avoidance of excess fluids after 6 h from randomization (amount of given fluids and balance over time up to 72 h will be registered and reported)
- Inotropic agents – to increase impaired flow, dobutamine is preferred – if ineffective, adrenaline may be used
- Glucocorticoids – recommended not to be used
- Blood products – red blood cell (RBC) transfusion trigger 70 g/L, unless ischemia or active bleeding [2]
- Renal replacement therapy (RRT) – suggested criteria according to conventional standard criteria [19, 20], with proven feasibility [21] as follows:
  - serum potassium ≥6.0 mmol/L, or
  - pH <7.20 and serum bicarbonate ≤10 mmol/L, or
  - evidence of severe respiratory failure, based on a PaO_2_/FiO_2_ < 200 and clinical perception of volume overload *and* oliguria, or
  - persistent severe AKI (serum creatinine remains >50 % the value recorded at randomization) for more than 72 h from randomization
- Lung-protective ventilation – positive end-expiratory pressure (PEEP) ≥5 cmH_2_O, tidal volume <8 ml/ideal body weight, and plateau pressure <30 cmH_2_O

### Inclusion criteria

Septic shock defined as (Appendix 1):

1. Infection (suspected or documented) *and*
2. Systemic mean blood pressure above 65 mmHg requiring any dose of vasopressors (norepinephrine, epinephrine, vasopressin) despite adequate fluid resuscitation (minimum of 20 ml/kg (actual body weight) crystalloids) *and*
3. Elevated lactate ≥3.0 mmol/L with suspected hypoperfusion

### Exclusion criteria

- Age below 18 or over 80 years
- Any other probable condition than sepsis affecting or expected to affect the central nervous system, including post cardiac arrest
- Myocardial ischemia
- Acute pulmonary embolism
- Terminal illness and not considered for full intensive care support
- Use of extra-corporeal membrane oxygenation (ECMO)
- Known liver disease – Child-Pugh classes B or C
- Known chronic kidney disease
- Known to be pregnant or lactating
- More than 4 h from fulfilled inclusion criteria (to be fullfilled at the latest 4 hours after ICU admission) to randomization (to be done in 8 hours from ICU admission)
- Other probable cause of hyperlactatemia
- Patients transferred from another intensive care unit (ICU)
- Patients with active hematological malignancy

### Randomization

Randomization will be done using a computer-based algorithm created by an independent statistician, to allow immediate and concealed allocation to the intervention arm. The patients will be stratified according to the site and presence/absence of chronic hypertension (with known medication). A varying block size will be used. Each patient will be allocated a unique patient ID-number. Randomization is targeted to be performed within 2 h after fulfillment of the inclusion criteria in the ICU (but no later than 4 h, the fulfillment of which is an exclusion criterion).

### Primary outcome measure

- Days alive in 30 days – with normal arterial blood lactate (first confirmed value of <2 mmol/L *and* without any inotropic or vasopressor agent)

### Secondary outcome measures

Time to normalization of lactate
Days alive with normal lactate (all values <2 mmol/L) in 30 days
Days alive without the use of inotropic or vasopressor agents in 30 days
Days alive without RRT in 30 days
Days alive without mechanical ventilation in 30 days
Days alive without any organ support (mechanical ventilation, RRT) in 30 days
New AKI according to the KDIGO classification (stages I–III)
8.. Days alive outside hospital in 90 days
Total amount of norepinephrine given until day 5
Number/total number of the following adverse reactions:
  - ventricular tachycardia/fibrillation
  - atrial fibrillation
  - myocardial infarction
  - skin necrosis
  - stroke
  - secondary bowel ischemia
  - limb ischemia
  - total numbers of serious adverse reactions (SAR) (numbers of patients and reactions)

### Exploratory outcomes

All-cause mortality at day 90.

### Blinding

Blinding of health care providers will not be feasible which infers that all clinical staff caring for the patients will be aware of the allocation during the intervention period. The two interventions may lead to different use of concomitant interventions, but the lack of blinding may also result in differences in the use of concomitant interventions during the intervention period. Hence, we will provide suggestions for the use of relevant co-interventions (see “Concomitant interventions”) and will record the use of them.

Information on the primary outcome and other secondary outcomes will be provided by the local investigators from patient charts, but the statistician doing the analyses will be blinded to which intervention the patients received. Information on whether the exploratory outcome of death occurs will be acquired through public registers (the National Civil Registries) without knowledge of which intervention group the patient was allocated to. In Switzerland, the patient or relatives will be contacted. The assessor of the outcomes will be blinded to the study group. The members of DSMB will remain blinded unless they request otherwise.

### Participant discontinuation and withdrawal

Patients who are withdrawn from the trial protocol will be followed up and analyzed as the remaining patients (intention-to-treat analysis, ITT).

### Suspension of the protocol

The protocol may temporarily be suspended for the individual patient, at the discretion of the treating clinicians, if the patient is to be resuscitated in the presence of any acute condition superimposed on septic shock, as judged by the investigator. Patients to be operated on at operational theatres may also have their procedures suspended during that time.

### Severe adverse reactions (SARs)

SARs to norepinephrine:

1. Cerebral hemorrhage seen on computed tomography (CT) or magnetic resonance imaging (MRI) scan
2. Cardiac arrhythmia resulting in the use of medication or electrical cardioversion
3. Psychiatric symptoms resulting in the use of antipsychotic drugs

Not registered as SARs to norepinephrine: tremor, headache, dizziness, and sweats are not registered. Hypertension is not registered. Dyspnea itself is not regarded as a serious adverse event (SAE). Hypersalivation, nausea, and vomiting are not registered.

SAEs will not be recorded as an entity, because the majority of septic ICU patients will experience several SAEs during their critical illness. The most important SAEs will be captured in the secondary outcome measures and in the daily Sequential Organ Failure Assessment (SOFA)-scoring. Patient charts will contain daily registrations of detailed clinical data, which can be obtained on request from the medical authorities. Trial investigators are to report suspected unexpected serious adverse reactions (SUSARs) without undue delay to the chief investigators, which in turn will report these to the Swiss, Finnish and Danish Health and Medicine Authorities within 7 days after the report has been received.

### Statistical plan and data analysis

#### Sample size and power

We assume 25 % mortality in both study groups, and this phase II trial will be unlikely to have the power to detect any mortality difference between the intervention groups. Therefore, a surrogate endpoint (days alive in 30 days with normal lactate and without vasopressors) combining survival, need for vasopressor therapy, and normalization of hyperlactatemia has been chosen. A post-hoc analysis from the FINNAKI study in a similar study population revealed a 30-day mortality rate of 25 %, and in 98 patients (of 128) who survived the mean time alive without vasopressors or hyperlactatemia within 30 days (720 h) was 650 h (SD 35) (unpublished data).

In the power analysis we assumed that the time with vasopressors or hyperlactatemia among the 30-day survivors follow a log-normal distribution with mean of 70 h (70 minus “treatment effect among survivors for the TTP group”) and SD 30, this simulated value was subtracted from 720 h (the number of hours in 30 days). For each configuration of sample size, mortality rate and expected treatment effect we simulated data and compared the two groups using a Mann-Whitney test. Ten thousand simulations were done for each configuration to compute the power. The required sample sizes (both groups combined) to achieve 80 % power as a function of mortality proportion (i.e., fraction of patients with a zero value for the primary endpoint) and average change in the primary endpoint among survivors are displayed in Table 1.

**Table 1:** Power calculations

	Change among survivors in hours (and among all patients)		
Mortality proportion	12 h (9 h)	18 h (13.5 h)	24 h (18 h)
20 %	340	156	90
25 %	438	194	118
30 %	562	270	158
35 %	660	348	210
40 %	660	456	282

The clinically relevant difference in the study primary outcome to be tested is 18 h (Amendment 1 to the study protocol – 26 October 2015). An adaptive sample size adjustment will be employed. After 150 patients have been included and followed for 30 days, the study statistician (Assoc. Professor T. Lange, University of Copenhagen) will redo the power calculation based on the observed mortality proportion and the observed dispersion in the primary outcome. The calculation will be done without un-blinding the study and will follow the previous recommendation by Chow and Chang (based on the revised power calculation a suggestion for a revised sample size will be presented to the investigators) (https://www.crcpress.com/Adaptive-Design-Methods-in-Clinical-Trials-Second-Edition/Chow-Chang/9781439839874).

### Statistical methods

**Fig. 1:**
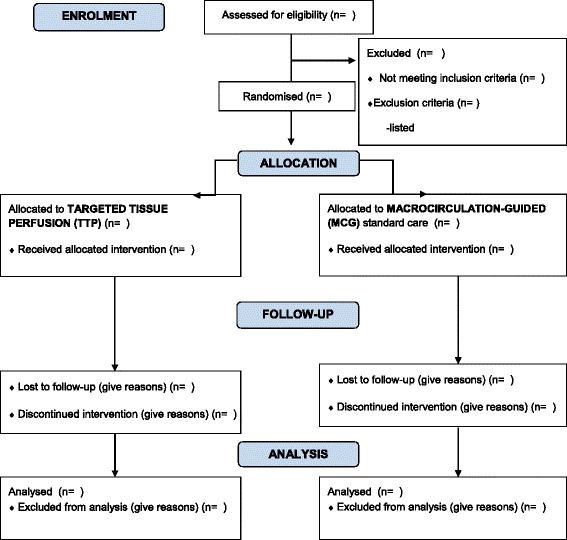
The TARTARE-2S study flowchart

The study flowchart is included as Fig. 1. A Mann-Whitney test for differences in continuous outcomes including the primary outcome measure, and Fisher’s exact test for dichotomous variables will be done as a primary analysis. A sensitivity analysis in subgroups of patients with and without previous hypertension will be performed. The components of the primary outcome measure (time to normalization of lactate, time to stopping of all vasoactive drugs, and day-30 mortality) are all included among secondary outcomes. In all tests a *p* value of less than 0.05 will be considered statistically significant. If missing data are >5 %, multiple imputations will be performed. A detailed statistical analysis plan is provided in Appendix 5.

### Interim analysis

As this trial is a feasibility trial we do not consider an interim analysis appropriate. As an interim analysis will not be performed, statistical early stopping criteria will not be applied. However, the Data Safety and Monitoring Board (DSMB) has full access to the safety data and will do a thorough review of these after 100 patients, and may recommend stopping the study anytime based on safety concerns.

### Intervention accountability

Every patient will be allocated a registration sheet to be kept in the site master file. The initials, birth date, screening number, time for randomization, and study arm will be included. The compliance of trial arm targets for each study patient will be checked hourly for the first 72 h, and clinical problems and given hemodynamic treatments will be registered until the study combined endpoint has been fulfilled, up to day 30. The originals for these documents will be kept as the study source data at each site.

### Registration

ClinicalTrials.gov: NCT02579525 (Registration date 19 October 2015).

### Data to be registered

**Fig. 2:**
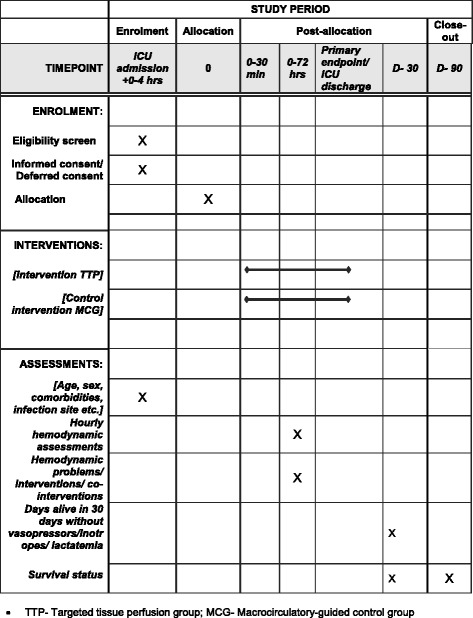
The schedule of enrollment, interventions, and assessments

The continuous data on hemodynamic parameters will be registered as 10-min medians using electronic PMDS, when possible and otherwise registered each hour to 72 h. For other data please see “Appendix 4.” The Standard Protocol Items: Recommendations for Interventional Trials (SPIRIT) figure regarding study interventions and timing is provided as Fig. 2.

### Data handling

Data will be entered into an electronic, web-based electronic Case Report Form (eCRF) from patient notes by trial personnel. Each patient will receive a unique trial identification number. Trial investigators will receive a personal username and passwords to access the TARTARE-2S trial web-page. Each site will only have access to site-specific data. Data will be handled according to the Swiss, Finnish and Danish laws. All original records (including Informed Consent Forms, eCRFs, and relevant correspondences) will be archived at trial sites for 15 years. The clean electronic trial database file will be delivered to the Inselspital Data Archive and maintained for 15 years and anonymized, if requested by the authorities.

### Monitoring

The trial will be externally monitored (Clinical Trial Unit, Bern) to Good Clinical Practice (GCP) standards. A centralized day-to-day monitoring of the eCRF will be done by the coordinating investigator.

### Ethical considerations

In addition to fluid therapy, norepinephrine administration to increase the MAP is a key element in the treatment of septic shock. As the intervention is very frequent, but not evidence-based and carries potential risks, we consider it to be in the wider interests of society and patients to perform research in this area, as clearly highlighted in a recent systematic review and meta-analysis [9]. The present trial is a phase II trial, i.e., a trial assessing both the feasibility of the protocol in a clinical setting and a patient-centered surrogate outcome. Should the trial prove feasible with separation between the two interventions, and suggest a benefit in terms of the primary endpoint, a larger phase III trial assessing 90-day mortality is intended. Thus, it is the opinion of the Steering Committee that this study is very important and ethically justified.

In some retrospective observational studies, low MAP has been associated with an increased risk of acute kidney injury (AKI) [22–24]. However, causal inferences cannot be drawn from observational data with the inherent limitation that the most severely ill patients inevitably have lower MAPs. In the present study: (1) a minimum safety limit of MAP has been set to the TTP group, (2) the study patients are carefully monitored and re-evaluated, and treatment goals are registered every hour. In addition, (3) the treating clinician should allow lower MAP values only in situations without any clinical problems, such as oliguria, in which cases individual use of higher MAP targets is allowed (and also in previously hypertensive patients). Thus, the Steering Committee has strong reasons to believe that the care of the study patients will be better and safer than the highly varying standard clinical practice; e.g., regarding fluid treatment [25]. In addition, high-quality randomized controlled trials (RCTs) focusing on unproven but widely used standard treatments have been shown to change clinical practice and to save future critically ill patients from untoward adverse events and death, e.g., by avoiding the use of starch in septic shock [26].

Treatment of septic shock is time-dependent and many patients will be unconscious and, due to fever and/or septic brain dysfunction, unable to consider an informed consent. Inclusion of only conscious less severely ill patients would cause a significant bias and jeopardize the study results. Furthermore, the treatment separation and effect are plausibly most obvious the earlier the randomization occurs. Of note, both treatment arms are within the frame of current standard clinical practice and no investigational drugs are used, thus, ethically allowing the potential use of a deferred consent. Therefore, patients will be included either with a deferred consent, or a proxy consent (next of kin) according to national laws in Finland, Switzerland, and Denmark (two-physician consent also allowed in Denmark). The patient or next of kin and/or general practitioner will be asked for a deferred consent if required by national law.

The trial will adhere to the trial protocol, the Helsinki Declaration in its latest form, GCP guidelines, and the national laws in the countries involved. Inclusion will start after approval by the ethics committees, medicines agencies, data protection agencies, and health authorities in the countries of the trial sites.

### Informed consent

The process leading to obtaining informed consent will be in compliance with all applicable regulations and national laws. Patients with septic shock are seldom able to consider their consent due to fever, mechanical ventilation, sedation, and/or septic encephalopathy. However, those patients who regain consciousness will be asked for informed consent as soon as possible. The possible benefit of the treatment (and the separation between the study arms) will plausibly be larger the sooner the treatment is started. In addition, it is important not to introduce a significant bias to the study results by only including less severely ill patients because most plausibly the most severely ill patients will benefit most. The consenting party will be provided with written and oral information about the trial so they are able to make an informed decision about participation in the trial. Written information and the consent form will be subjected to review and approval by the National Ethics Committee.

### Duration

For clinical treatment, primary and secondary endpoint up to 30 days; follow-up exploratory endpoint until day 90.

### Co-enrollment

Possible simultaneous co-enrollment to any other RCT will be discussed by the Management Committee.

### Timeline

Trial sites determined – September 2015

Governance approval applications submitted – October 2015

First participant enrolled – April 2016

Last participant enrolled – November 2017

Follow-up completed – December 2017

Data analysis and submission for the main publication – March 2018

Laboratory analyses completed – June 2018

Publications of clinical sub-studies and laboratory analyses completed – December 2018

### Trial organization

#### Steering Committee

Ville Pettilä, Helsinki University Hospital, and Inselspital, Bern (CI1)

Jukka Takala, Inselspital, Bern, Switzerland (CI2)

Stephan Jakob, Inselspital, Bern, Switzerland

Anders Perner, Copenhagen University Hospital, Rigshospitalet, Denmark.

#### Site principal investigators (PIs)

Tobias Merz, Inselspital, Bern

Erika Wilkman, Helsinki University Hospital

Sari Karlsson, Tampere University Hospital

Anders Perner, Rigshospitalet, Copenhagen

#### Data Safety and Monitoring Board (DSMB)

Professor Konrad Reinhart (chair), Jena

Professor Peter Jüni, Bern/Toronto

Professor Jan Wernerman, Stockholm

#### Independent statistician

Assoc. Professor Theis Lange, Centre for Research in Intensive Care and Section of Biostatistics, University of Copenhagen.

#### Trial sponsors

The coordinating investigators, CI1 Ville Pettilä and CI2 Jukka Takala, are trial sponsors.

### Laboratory measurements

Ethylenediaminetetraacetic acid (EDTA) plasma samples and whole blood will be drawn as soon as possible after ICU admission, and at 72 h from ICU admission for later analysis of patient-related changes in biomarkers indicating endothelial/myocardial damage, such as CD73 and vascular adhesion protein-1 (VAP-1), heparin-binding protein (HBP), chromogranin A (CgA), metabolomics, and mitochondrial function tests.

### Publication plan

All trial results whether positive, negative, or neutral will end up in the public domain, preferably in a peer-reviewed publication. All trial sites with at least 25 randomized patients will be granted one authorship for the site PI, and each additional 25 patients will guarantee one additional authorship for the site AIs. A trial statistician (TL) will also be granted an authorship. The order of authorships for the study main publication will be as follows: VP, SJ, and AP will be the first, second, and third, and JT the last author. Other authors will be in the order of included number of patients with high-quality data: site PIs first, and AIs thereafter. The authorships for the publications of the main and sub-studies will follow the International Committee of Medical Journals Editors’ (ICMJE) principles. The full protocol and the participant-level anonymized dataset will be submitted as appendices with the main paper to be publicly available after the study publication. The SPIRIT checklist for clinical trial protocol is provided as Additional file 1.

### Perspectives

Septic shock affects millions of patients worldwide annually. The results of study will – in any case – have a major influence on the clinical management/international guidelines regarding treatment of septic shock. If supporting our hypothesis of the beneficial effects of TTP, the current recommendations will have to be changed leading to major scientific and societal impacts on heath and health care costs. In case MCG proves to be better, the study results will for the first time confirm the widely recommended minimum MAP level. Given that several different pathophysiological pathways are probably in interaction and may only partly explain the clinical course of the disease, the potential strength of measuring several biomarkers/mitochondrial function/gene expression and protein synthesis in the sub-studies of this phase II study, will give us the opportunity to compare their relative importance and predictive power with regard to organ dysfunction.

## Discussion

Conduct of the TARTARE-2S trial is in broad agreement with the international guidelines [7] regarding the control group. The targeted patient population fulfills the recent Sepsis-3 septic shock definition [27], representing the most severely ill septic shock patients with plausibly the highest possible chance to show a difference between the intervention arms [28], if such a difference exists. The experimental approach is supported by a recent review [9] highlighting the paucity of evidence to support the guidelines and additional evidence suggesting the relevance of more detailed monitoring of peripheral perfusion [13, 14] to guide the treatment in septic shock. The TARTARE-2S trial may be one of the first trials to bridge the gap between evidence and clinical practice and to provide detailed data on efficacy and safety of TTP to guide treatment in patients with septic shock.

## Trial status

Patient recruitment has started in May 2016. Ethics and hospital approvals have been granted in Finland, and in Switzerland, and have been applied in Denmark (as of July 28, 2016).

## Appendix 1 The trial criteria for septic shock

*Septic shock* is defined as:

1. Infection (suspected or documented) *and*
2. Systemic mean blood pressure above 65 mmHg requiring any dose of vasopressors (norepinephrine, vasopressin) despite adequate fluid resuscitation (minimum of 20 ml/kg (actual body weight) crystalloids) *and*
3. Elevated lactate ≥3.0 mmol/L with suspected hypoperfusion

## Appendix 2

**Table 2:** The trial targets for the treatment arms. (Register hourly up to 24 h (every 2 h thereafter until study endpoint using tick boxes for targets)

I. Intervention group – targeted tissue perfusion (TTP) care:	
Primary targets	
Capillary refill time (CRT)/every hour	<3 s
Skin mottling [17]/every hour	Absent
Peripheral temperature/every hour	Warm
Urine output/every hour	≥0.5 mL/kg/h
Arterial lactate [7]/per 2 h	<2.0 mmol/L
Mean arterial pressure (MAP)	50–65 mmHg (minimum as a safety limit)
^a^If previous hypertension [7]	^a^65–70 mmHg
^b^If oliguria <0.3 ml/kg [7]	^b^2-h trial 75–80 mmHg,
	If diuresis improves, continue for 2 h and re-evaluate
Secondary target	
Continuous SvO_2_ [7], if available	>65 %^c^
II. Control group – macrocirculatory targets-guided (MCG) standard care	
Primary targets	
Mean arterial pressure (MAP) [7]	65–75 mmHg
^a^If previous hypertension [7]	^a^75–80 mmHg
^b^If oliguria <0.3 ml/kg	^b^2-h trial 85–90 mmHg
	If diuresis better, continue for 2 h and re-evaluate
Central venous pressure (CVP) [7]	8–12 mmHg
	Adequate fluid therapy is indicated to restore clinical hypovolemia up to the recommended CVP level of 8–12 mmHg, if needed
Urine output [7]	≥0.5 mL/kg/h
Secondary target	
Continuous SvO_2_ [7], if available	>65 %^c^

Dellinger et al. [7] according to the SSCG – Surviving Sepsis Campaign Guidelines: MAP, CVP, diuresis, SvO_2_ – 1C, lactate – 2C (1 – a recommendation, 2 – a suggestion, C – low level of evidence)

^a^Higher MAP targets may be required for septic shock patients with previous hypertension; ^b^and a test of providing higher MAP target for 2 h is recommended for those with oliguria

^b^The treating physicians should target to the lowest possible vasopressor use to maintain the highlighted lowest possible MAP level in each treatment arm; however, allowing individual higher MAP targets with specific reasons

^c^Measuring of ScVO_2_ is not recommended [1, 4, 5]. If monitoring is clinically required, use of a pulmonary artery catheter (PAC) is recommended. Pulse continuous cardiac output (PICCO) may be used for thermodilution cardiac output measurements

## Appendix 3

**Table 3:** Data to be gathered regarding each hemodynamic target and treatment decision/change. Indicate when treatment start or change (at the same time)

(a). Hemodynamic problem(s)	
Hemodynamic problem(s)	Tick
Hypovolemia	
Hypervolemia	
Inadequate flow/cardiac index (CI)	
Tachycardia	
Inadequate contractility	
Inadequate afterload/hypotension	
Excessive afterload/hypertension	
Excessive vasopressor dose	
(b). Given treatment(s)	
Treatment(s) given	Tick
Volume	
Diuretics	
Inotrope	
Inotrope decrease	
Inotrope increase	
Vasopressor increase	
Vasopressor decrease	
Beta-blocking agent	
Vasodilating agent	

## Appendix 4 Data to be registered

Data will be obtained in eCRFs from a combination of national registers and medical records. Bedside target list for treatment reasons and decisions will be implemented in the PDMS (*) at each site, if possible.

Baseline variables

- National identification number (if available)
- Sex
- Age at randomization
- Estimated height
- Measured (if not possible estimated) weight
- Comorbidities? Y/N (hypertension*, chronic heart failure*, previous myocardial infarction, previous stroke, chronic obstructive pulmonary disease*, diabetes*, hematological malignancy*, metastatic cancer*, AIDS*)
- Elective or emergency admission*
- Operative or non-operative admission*
- Site of infection (pulmonary/abdominal/urinary tract/soft tissue/other)
- Previous plasma/serum creatinine (7–365 days before hospital admission), if available

24-h from ICU admission:

- Values for Simplified Acute Physiology Score (SAPS II)*

Twenty-four hours before randomization

- Volume of resuscitation fluids (crystalloids, colloids and blood products specified in milliliters)
- Hemoglobin (lowest)*
- SOFA score *variables**

At randomization

- Norepinephrine dose and mean arterial pressure (MAP) at the same time
- Total fluids given in last 2 h
- Lactate (last value from the previous 2 h)

Continuously as 10-min medians in the first 72 h after randomization:

- Systolic arterial pressure*
- Mean arterial pressure*
- Central venous pressure*
- Heart rate*
- Peripheral oxygen saturation (SpO_2_)*
- Continuous central venous oxygen saturation, if available
- Continuous mixed venous saturation, if available
- Dose of norepinephrine*
- Dose of dobutamine*

One-hour intervals (for 72 h)

- Urinary output*
- Volume of given fluids
- CRT – only in the TTP group
- Assessment of upper arm temperature – only in the TTP group
- Assessment of skin mottling score – only in the TTP group

Two-hour intervals (for 72 h unless study endpoint reached)

- Arterial lactate measured in both groups

Four-hour intervals during the first 72 h after randomization (if not discharged from the ICU)

- Arterial blood gas tensions (PaO_2_, PaCO_2_), pH and base excess (BE)
- Calculated Mixed v-aCO_2_/Da-vO_2_-ratio (if pulmonary artery catheter, PAC)
- Cardiac index (if PAC)
- Stroke index (if PAC)

Daily in the first 7 days after randomization (if not discharged from the hospital):

- SOFA score variables*
- Total volume of fluids
- Estimated fluid balance
- Dose of glucocorticoids (not recommended)

Daily up to 30 days:

- On mechanical invasive- or non-invasive ventilation? Y/N
- Ventilator settings at 8 a.m. (PEEP, Pplat, Vt)
- Use of muscle relaxants during the last 24 h? Y/N
- Presence of acute kidney injury (AKI) according to KDIGO classification? Y/N/and stage (I–III)
- Use of renal replacement therapy? Y/N
- Use of vasopressors? Y/N
- Use of inotropic agents? Y/N
- Ischemic events
- SARs and SUSARs

## Appendix 5 A detailed study statistical analysis plan

The primary analysis of the primary outcome (as well as secondary outcomes b, c, d, e, f, h, i, and j) will be an unadjusted Mann-Whitney test. As a sensitivity analysis the *p* values will be recalculated using permutations within groups defined by site and the presence/absence of hypertension. This test is a non-parametric version of an ANOVA adjusted for site and presence/absence of hypertension. A total of 100,000 permutations will be conducted for each test. The effect sizes will be quantified by direct comparisons of the raw means between groups. Confidence intervals will determined by non-parametric bootstrapping to accommodate the highly non-normal distribution of the outcomes.

The secondary endpoint “a” will be assessed by a Cox model treating death before normalization of lactate as a competing event. The Cox model will be adjusted for site and presence/absence of hypertension. The effect size will be quantified using the adjusted hazard ratio.

The secondary outcome “g” will be assessed using Fisher’s exact test. Effect sizes will be analyzed using crude proportions along with standard normality approximation-based confidence intervals.

The exploratory endpoint (90-day survival) will be assessed using Fisher’s exact test. Effect sizes will be quantified using odds ratios from a logistic regression adjusted for site and the presence of hypertension. As a sensitivity analysis a Cox model of time to death will also be employed. The Cox model will be adjusted for site and the presence/absence of hypertension. Here, the effect size will be quantified using the adjusted hazard ratio. The assumption of proportional hazards will be assessed using Schoenfeld residuals. Finally, Kaplan-Meier plots will be produced.

If any of the tests presented above must be based on data with more than 5 % missing multiple imputation will be employed. If just one test needs to be based on multiple imputation all tests will be conducted both as complete case analysis and multiple imputation. However, only for tests based on data with more than 5 % missing will the multiple imputation approach constitute the primary analysis. The multiple imputation will be conducted in R using the mice package and the recommendation in accordance with recommendations in the documentation of this package. All outcome variables (secondary and primary), sex, and age as well as stratification and treatment variables will be included in the multiple imputations procedure.

In all tests a *p* value of less than 0.05 will be considered statistically significant. It is noted that as the adaptive sample size recalculation is not depended on a particular value of the treatment effect at the time of recalculation, there is accordingly no need to adjust the significance level to take a possible adjustment of sample size into account. All analyses will be conducted in the statistical software package R. The full statistical analysis will be conducted blinded to actual treatment allocation and a full blinded statistical report produced. Of note, un-blinding will first happen after the full statistical report has been finalized.

## Additional file

Additional file 1: SPIRIT checklist. (PDF 2231 kb)
